# Editorial

**Published:** 1869-06

**Authors:** 


### Editorial.

Illinois State Medical Society.—The proceedings of the recent annual meeting of this Society must be deferred until the July number of the Examiner.

Meeting of the American Medical Association at New Orleans.—The record of proceedings of the recent annual meeting of this great national organization occupies so much space in the present number that our clinical matter and book notices are both crowded out. And, yet, our readers will get but a very imperfect idea of the work done by the Association during its recent session. Nearly all the scientific part of the work was done in the Sections, where many papers and reports were read and discussed, very much to the pleasure and profit of those in attendance. But of all these, nothing appears in the record of proceedings as published. We notice, also, that many of our cotemporary journals are publishing the record literally as it was reported in the New Orleans daily papers, and are consequently giving currency to some errors, especially in regard to committees. For instance, in several, the President, Dr. W. O. Baldwin, is represented as appointing himself at the head of the Committee on the establishment of "a National Medical College," while, the truth was, that after he had announced the committee, his own name was added by a special vote of the Association. Again, in another part of the record, they represent the President as *adding to the Committee on Correspondence with Medical Societies*, Drs. Davis, Wetherly, and Toner; while the true reading should be, that the gentlemen named were appointed a committee, charged with the special duty of presenting to the several State medical societies a series of resolutions that had been already adopted by the Association. The recent meeting was one of the most pleasant and profitable that we have attended.

Dr. Baldwin presided with ability, and all the business of the Association was transacted wlth unusual harmony and facility. The hospitalities received were abundant, and of the most appropriate character. The week of our sojourn in the Crescent City will ever bring to mind only the most pleasant memories.

---

Association of American Medical Editors.—In the present number of the Examiner, we publish the proceedings taken in the formation of a permanent organization of those connected with the medical press of our country.

The objects of the organization as set forth in the proceedings are important, and, if judiciously pursued, will lead to the most important results.

That mutually advantageous arrangements can be made by associated action, in reference to foreign exchanges, there can be no doubt. That annual meetings will be productive of more unity of action, and, consequently, greater influence, and far greater attention to some topics of permanent importance to the profession, is equally evident. The medical periodical press should be, not merely a medium for the dissemination of knowledge in the various departments of medical science, but it is preëminently the proper and only *efficient* medium through which to reach and influence the general sentiment of the profession, on all questions relating to medical education and medical ethics. It has often been said that many of the medical periodicals are published by and constitute simply the organs of the faculties of particular medical colleges, and hence they cannot be relied on to expose abuses or to efficiently advocate independent measures of improvement. This may be true in some instances, but association and consort of action will do much to obviate that evil, and develop much more fully individual editorial responsibility and influence. Hence, we hope to meet, next May, in Washington, representatives from every regular medical periodical in our country. In the meantime, let our editorial brethren commence the work at once of discussing candidly, courteously, and thoroughly the all-important topic of medical education, in all its relations. If the honor and usefulness of the profession, as well as the nature of the medical sciences, require a fair degree of preliminary education and mental discipline, on the part of those who commence the study of medicine, let us not only say so, but let us continue to discuss the subject until some practicable method for accomplishing the object is actually adopted. What shall be the standard required? Shall its enforcement be required by the medical colleges, or by the private preceptor, through boards of censors, appointed by the local medical associations? These are the questions. If the lecture terms in our colleges, generally, are too short to permit an adequate course of instruction? if the system of giving heterogeneous instruction on all the branches of medical science and practise, to students in all stages of study in one class, is contrary to the dictates of common sense, and productive of many evils? and if the making of the college diploma a license to practise and a full admission into the profession tends directly and strongly to keep the competition and rivalry among the schools, on the basis of the shortest terms and smallest possible requirements for the degree? let the facts be kept constantly before the whole reading portion of the profession, until a united and enlightened sentiment devises and enforces the proper remedies. That the social status and usefulness of a great profession, embodying the highest attainments in science and the most humane objects, should be injured, year after year, and generation after generation, by manifest and acknowledged defects in its system of education, is simply puerile.

For us to continue listening to annual addresses of presiding officers of social organizations, whether state or national, and the reports of committees, reciting in glowing colors the defects, absurdities, and practical evils of such system, as we have been doing for twenty years past, without agreeing upon some practical measure of improvement and setting ourselves earnestly about the work of executing them, is to make ourselves the laughing-stock of all intelligent men. The only medium through which the great mass of the profession in all parts of the country can be reached is the medical periodical press. Let those having the editorial management of that press realise their responsibility and act accordingly.

---

ILLINOIS STATE MICROSCOPICAL SOCIETY.—A society with this title has been recently organized in this city, under a charter granted by the Legislature of the State. Its meetings thus far have given promise of great usefulness. Dr. W. W. Allport is the President, and James Hankey, Esq., is Corresponding-Secretary. We shall notice some of the doings of this Society more at length hereafter.

---

MEDICAL LAW.

THE State of Minnesota has shown a very commendable example to many of her older sisters in passing a law to protect her people from empiricism and imposition in the practice of medicine and surgery. The bill is so much to the point, and so commendable in its provisions as regards protection of the rights of legitimate medicine, that we give it entire, taking the occasion to commend it to the special attention of all parties concerned:—

SECTION 1. That it shall be unlawful for any person within the limits of said State, who has not attended at least two full courses of instruction, and graduated at some school of medicine within the United States, or of some foreign country, or who cannot produce a certificate of qualification from some State, district, or county medical society, and is not a person of a good moral character, to practise medicine in any of its departments, or perform any surgical operations for reward or compensation, or attempt to practise medicine, or prescribe medicines, or perform any surgical operation for reward or compensation, within the said State of Minnesota.

SEC. 2. Any person living in the State of Minnesota, or any person coming into said State, who shall practise medicine, or attempt to practise medicine, in any of its departments, or perform, or attempt to perform, any surgical operation upon any person within the limits of said State, in violation of Sec. 1, of this Act, shall, upon conviction thereof, be fined not less than fifty dollars, nor more than one hundred dollars for such offence; and upon conviction for a second violation of this Act, shall, in addition to the above fine, be imprisoned in the County jail of the County in which such offence shall have been committed, for the term of thirty days; and in no case wherein this Act shall have been violated, shall any person so violating, receive a compensation for services rendered: *Provided*, nothing herein contained shall, in any way, be construed to apply to any person practising dentistry exclusively.

SEC. 3. No person who fails or neglects, on or before the first day of October, 1869, to file, in the office of the Clerk of the District Court of the County in which he resides or keeps his office, a sworn copy of the certificate or diploma of some school or college of medicine, that he has attended at least two full courses and graduated at such school, or a sworn copy of a certificate of qualification of some State, District, or County medical society, shall be permitted in any court of this State to sue for or recover any compensation for his services, advice, or attendance as a physician or surgeon; and the failure to file a sworn copy of such diploma or certificate, as above provided, shall be *prima facie* evidence that he has not attended or graduated at any school of medicine, or received a certificate of qualification from any State, District, or County medical society.

SEC. 4. Any person studying medicine with a preceptor, qualified as in this Act above provided, shall have three years from the commencement of his term of study to comply with the provisions of this Act.

SEC. 5. This Act shall take effect and be in force from and after the first day of October, 1869.

We are indebted to Dr. Samuel Wiley, of St. Paul, Minn., for the above copy of the bill. It was passed March 4th, 1869. —*The Medical Record.*

---

NOTE ON THE CURE OF ACUTE ORCHITIS IN TWENTY-FOUR HOURS.—By FURNEAX JORDAN, F.R.S., Surgeon to the Queen's

Hospital, Professor of Surgery at the Queen's College.—It is gratifying to me to know that Mr. Noble Smith has found "most satisfactory" results (*British Medical Journal*, Jan. 30th) from the treatment of acute orchitis which I described at Oxford. May I suggest that still better results may be obtained by using a solution of nitrate of silver and applying it *immediately* the cases present themselves?

In very acute cases, I add a little vesication over the femoral artery of the same side. This is the treatment, as seen in a severe double orchitis treated in my absence, and reported to me by a correct observer, our house-surgeon, Dr. Jolly. A man, aged 30, had intense pain, intolerable tenderness, and great swelling and induration in both testicles, and could not stand upright. The scrotum was covered with a solution of nitrate of silver (two drachms to an ounce); a stripe of vesication was established over the upper halves of both femoral arteries by means of linamentum iodidi; and the testicles supported with cotton-wool. He was well in 24 hours.

The treatment of orchitis is of more than ordinary importance, from the discovery by Dr. Marion Sims that closure of the vas deferens from acute orchitis is a common cause of sterility—often where the blame is laid to the wife.

The return of some urethral discharge is best removed, often in a few days, by maintaining, with iodine, a disc of milder counter irritation, the centre of the disk being the genital organs. On this principle I treat all inflammations of the genito-urinary organs, male and female; adding in the acute form a little vesication on the sheltered position of the femoral arteries.

The above treatment of orchitis is simply an illustration of a new system of treating all inflammatory diseases, and which I constantly adopt in all, with a success proportionately as great as in acute orchitis. A brief sketch of the system appears in the February *Practitioner.—British Med. Jour., and New Orleans Jour. of Medicine.—N. Y. Medical Gazette.*

---

Creasote in Typhoid Fever.—M. Pécholier, of Montpellier, has recently been conducting a series of interesting researches on the action of creasote in typhoid fever. Conceiving the disease to be one, *totius substantiæ*, depending on certain changes in the blood, caused by the action of an organized ferment, which draws from the blood the materials necessary for its nutrition, and exhales those thrown off by its decomposition, M. Pécholier has been led to employ creasote as an antifermentive agent. Sixty patients at the Hôpital St. Eloi were chosen as the subjects of the experiment. Every day a draught containing three drops of creasote, two of essence of lime, ninety grammes of water, and thirty grammes of orange-flower water was administered to the patients. At the same time, enemata were given, containing from three to five drops of creasote. M. Pécholier states, as the result of his experiments, that creasote employed in weak doses, either in draughts, injections, or in the form of vapor, at the outset of typhoid fever, acts powerfully in diminishing the intensity of the disease, and shortening its duration. M. Pécholier adds that the employment of the remedy as a prophylactic agent in schools, garrisons, hospitals, etc., during epidemics, would be of extreme efficacy.—*Lancet.*—*N. Y. Med. Gazette.*

---

SUBCUTANEOUS MEDICATION FOR SYPHILIS.—Dr. Max Van-Mons has reported in the Brussels Academy five cases of secondary syphilis, with indurated chancres, treated by the subcutaneous injection of calomel. Three of the cases received two or three injections each (quantity not stated), at intervals of about 12 days. At the time of writing, these were nearly or quite cured, after the lapse of 37, 20, 19 days respectively; and the remaining two cases, which had each received one injection, only eight days previously, were notably improved. If this discovery is verified, it will prove one of the most important ever made in regard to the treatment of syphilis.—*Wiener Med. Wochenschrift*, No. 24. D. F. L.—*Med. and Surg. Jour.*

---

DEATH OF DR. ALEXANDER H. STEVENS.—At a special meeting of the New York Academy of Medicine, held April 2d, 1869, for the purpose of paying a tribute to the memory of the late Alexander H. Stevens, M.D., a series of resolutions were unanimously passed, expressive of the high estimation in which he was held by the members of the Academy, and of their deep sorrow at his departure.—*Boston Med. and Surg. Journal.*

